# Identification of anti-mouse PD-1 agonist antibodies that inhibit T cell activation

**DOI:** 10.3389/fimmu.2025.1631929

**Published:** 2025-09-23

**Authors:** Lingling Liu, Shoma Takashima, Yosuke Tokumaru, Naoko Ikuta, Yuka Nakajima, Akio Ohta

**Affiliations:** ^1^ Department of Immunology, Institute of Biomedical Research and Innovation, Foundation for Biomedical Research and Innovation at Kobe, Kobe, Japan; ^2^ R&D Division, Drug Discovery Department, Meiji Seika Pharma Co., Ltd., Tokyo, Japan

**Keywords:** immunosuppression, cancer immunotherapy, autoimmune diseases, immune checkpoint, Fc receptor

## Abstract

PD-1-stimulating agents have been projected to be unique immunosuppressants for the treatment of undesirable inflammatory conditions including autoimmune diseases. We recently characterized anti-human PD-1 (hPD-1) agonist antibodies, which showed a significant immunosuppressive effect in hPD-1 knock-in mice. However, the lack of anti-mouse PD-1 (mPD-1) agonist antibody has been a limitation in testing the efficacy of PD-1-targeted therapy using various disease models. To find mPD-1 agonist antibody, we assessed biological activities of commercial anti-mouse PD-1 mAb clones. Agonist activity was evident in RMP1-30, which did not block PD-1-PD-L1 interaction. In contrast, 29F.1A12 was the exceptionally strong blocking antibody. Interestingly, RMP1–14 was a dual-function antibody offering decent blocking activity and the agonist activity comparable to RMP1-30. In this assessment, PD-1 expression levels critically affected both blocking and agonist activities. T cells expressing PD-1 at higher levels were stronger responders in the agonist assay, while cells with lower PD-1 expression were more sensitive in detecting blocking activities. Considering physiologically-relevant PD-1 levels, RMP1–14 would substantially behave as a blocker *in vivo* consistent with its frequent use to enhance anti-tumor immunity. Taken together, RMP1–30 may be useful as mPD-1 agonist antibody although its *in vivo* efficacy may vary dependent on the local Fc receptor availability and PD-1 levels on target cells. It should be also noted that *in vivo* use of this rat IgG2b clone may require attention to the mechanism of immunosuppression that may involve PD-1 agonism and the depletion of PD-1-expressing effector cells.

## Introduction

Endogenous immunomodulatory mechanisms are the targets of therapeutic intervention to augment or attenuate inflammatory response (1). One of the most notable success is PD-1-targeted cancer immunotherapy. The immunosuppressive activity of PD-1 serves as a negative feedback mechanism to prevent unnecessary immune activities. The significance of PD-1-dependent immunoregulation has been demonstrated by spontaneous autoimmunity in PD-1-deficient mice (2–4). The blockade of PD-1 interaction with its ligand PD-L1 effectively enhanced anti-tumor immunity and improved therapeutic outcome in human patients (5, 6).

The success of cancer immunotherapy by PD-1 blocking Abs indicated that the biological impact of PD-1-targeted intervention is strong enough to change the course of inflammatory outcomes in the human immune system. In addition, since PD-1 regulates the functions of T cells (7, 8), NK cells (9, 10) and B cells (11, 12), PD-1-targeted immunotherapy is expected to be versatile to modulate both cellular and humoral immune responses. With these reasons, PD-1 stimulation may be an effective treatment of inflammatory disorders including autoimmune diseases where downregulation of overwhelming inflammatory response is desirable (13, 14).

For the pharmacological stimulation of PD-1, various types of PD-L1-based agonist molecules and anti-PD-1 agonist antibodies have been proposed as a new class of immunosuppressant. PD-L1-based stimulators include PD-L1-Fc (15) and PD-L1-T cell receptor fusion proteins (16). Designed agonist protein mimicking PD-L1 was also generated (17). An alternative approach is to interrupt PD-L1-CD80 interaction and set PD-L1 free from the inactive CD80-bound form (18). Anti-inflammatory effect has been reported with several anti-PD-1 antibody clones (19–22). Such antibodies are often referred as agonists solely because of the immunosuppressive outcome after *in vivo* injection; however, their mechanism of action was unclear. Indeed, it should be noted that anti-PD-1 mAbs might be able to downregulate inflammation by the depletion of target cells. Since pathogenic immune effector cells express PD-1, their depletion has been shown to stop proinflammatory activities (23–26). Therefore, it was important to establish anti-PD-1 antibodies that can stimulate the immunosuppressive activity of PD-1.

Our previous screening of anti-hPD-1 mAb panel identified a group of PD-1 antibodies, which could trigger the immunosuppressive PD-1 signaling (27). The agonist antibodies indicated characteristic recognition of the membrane-proximal extracellular region of PD-1 molecule. This trend was in sharp contrast to blocking antibodies, which specifically find the membrane-distal PD-L1-binding domain as their binding site. Another requirement for the agonist activity was PD-1 ligation through the antibody interaction with Fc receptor on the surface of antigen-presenting cells. Subsequent studies by others provided further evidence for these requirements to PD-1 agonist antibodies (28, 29).

Immunosuppressive efficacy of PD-1 agonist mAbs *in vivo* implicated its promise as a new class of immunosuppressant (27). However, anti-PD-1 mAbs with proven agonistic activity are all against hPD-1 so far and are not compatible in murine disease models unless immune cells are made to express hPD-1. The unavailability of anti-mPD-1 agonist mAb hampers the analysis in different types of inflammation using mice in various genetic background and gene manipulation. To overcome this limitation, we examined agonist activity in commercially available anti-mPD-1 mAb clones and found that RMP1–30 is an agonist antibody. Surprisingly, RMP1-14, which has been frequently used as a blocker, also had an agonistic activity with the recognition of membrane-proximal region. Recent studies reported some agonistic activities in PD-1 blocking antibodies, nivolumab and pembrolizumab (28, 29), although our screening of anti-hPD-1 mAb panel identified no mAb clone with notable dual activities (27). We also discuss *in vivo* role of such antibodies with dual activities.

## Materials and methods

### Cell lines

Parental DO11.10 T cell hybridoma and IIA1.6 B lymphoma cells were provided by Dr. Tasuku Honjo (Kyoto University). IIA1.6 cells are capable of presenting the cognate antigenic peptide, OVA_323-339,_ to DO11.10 cells but lack PD-L2 and Fc receptor expression (**Supplementary Figure 1**). PD-1-deficient DO11.10 T cell hybridoma and PD-L1-deficient IIA1.6 cells were from Dr. Taku Okazaki (Tokyo University). These cells were maintained at 37°C, 5% CO2 in RPMI1640 medium containing 10% fetal bovine serum, 6.25 mM HEPES, 2.5 mM L-glutamine, 0.625 mM sodium pyruvate, 0.625x non-essential amino acid solution, 62.5 μM 2-mercaptoethanol, 125 U/ml penicillin, 125 μg/ml streptomycin and 6.25 μg/ml gentamycin. HM266, a hybridoma clone producing anti-human PD-1 antibody, was originally established by Drs. Satoshi Nagata and Haruhiko Kamada (National Institutes of Biomedical Innovation, Health, and Nutrition).

### Retroviral transduction

Retroviral plasmids containing wild-type mPD-1, mPD-1(hu_38-48_) or mFcγRIIB were generated by inserting cDNA into MSCV-IRES-Thy1.1 DEST (Addgene; cat#17442). These plasmids were transfected to Plat-E cells (Cell Biolabs) using FuGENE HD (Promega; cat#E2311). After the extrusion through a 0.45-μm Minisart syringe filter (Sartorius; cat#16533), the retroviral supernatant was centrifuged for 2 hours at 32 °C in a culture plate coated with RetroNectin (Takara Bio, cat# T100A). Centrifugation was repeated after loading the cells to the retrovirus-coated culture plate at 800*g* for 10min at 32 °C. After the stable delivery of mPD-1 gene, DO11.10 cells expressing mPD-1 at high levels (mPD-1^high^) and at intermediate levels (mPD-1^int^) were sorted using FACSMelody Cell Sorter (BD Biosciences). The expression of mPD-L1 and mFcγRIIB in the gene-transduced IIA1.6 cells was confirmed by flow cytometry (**Supplementary Figure 1**).

### Antibodies

Purified anti-mPD-1 mAbs we tested were RMP1-30 (rat IgG2b; Leinco Technologies, cat#C3442), 29F.1A12 (rat IgG2a; Biolegend, cat#135246), RMP1-14 (rat IgG2a; Biolegend, cat#114110) and J43 (hamster IgG; eBioscience, cat#16-9985-85). See **Supplementary Table 1** for other antibodies used in this study. Anti-hPD-1 agonist mAb HM266 were produced by culturing the hybridoma cells in CD hybridoma medium (Gibco, cat#11279023) supplemented with 8 mM L-glutamine, 20 U/ml penicillin, and 20 μg/ml streptomycin in a CELLine bioreactor flask (Duran Wheaton Kimble, cat#WCL1000).

### PD-1 blocking activity and agonist activity

DO11.10 cells (5 × 10^4^ cells) were stimulated with OVA_323–339_ peptide (2 μg/ml; Eurofins Genomics) in the presence of IIA1.6 cells (1 × 10^4^ cells) for 15 hours. IL-2 levels in the culture supernatants were determined using mouse IL-2 DuoSet ELISA (R&D Systems, cat#DY402). The blocking activity of anti-mPD-1 mAbs was determined by the reversal of PD-L1-dependent immunosuppression in the co-culture of mPD-1^+^ DO11.10 cells and mPD-L1^+^ IIA1.6 cells. For the agonist activity, DO11.10 cells expressing wild-type mPD-1 or mPD-1(hu_38-48_) were co-cultured with PD-L1^-^ mFcγRIIB^+^ IIA1.6 cells, and IL-2 reduction by the addition of anti-mPD-1 mAb was monitored.

### Competitive inhibition of PD-L1-Fc binding

DO11.10 cells expressing mPD-1 were preincubated with anti-mPD-1 mAb (5 μg/ml) for 15min, and subsequently incubated with mPD-L1-Fc (10 μg/ml; Biolegend, cat#758206) for 15min. After washing, cells were further incubated with PerCP-Cy5.5-labeled anti-human IgG Fc mAb to detect mPD-L1-Fc binding. The extent of PD-L1-Fc binding was analyzed using LSRFortessa X-20 and FlowJo (BD Biosciences).

### Stimulation of T cells from DO11.10 mice

DO11.10 T cell receptor-transgenic mice were obtained from the Jackson Laboratory (Bar Harbor, ME). Mice were maintained under specific pathogen free condition in the animal facility at the Institute of Biomedical Research and Innovation. Animal experiments were conducted in accordance with the protocol approved by the IACUC of Foundation for Biomedical Research and Innovation at Kobe. OVA_323–339_ peptide (2 μg/ml) were added to freshly prepared spleen cells from DO11.10 mice. Activated CD4^+^ T cells were maintained with mouse IL-2 (2 ng/ml; Peprotech, cat#212-12) since the second day of culture. On day 8, T cells (2 x 10^5^ cells) were restimulated with ovalbumin (100 μg/ml; Fujifilm Wako Pure Chemicals, cat#012-09885) in the presence of IIA1.6 cells (1 x 10^5^ cells). To evaluate the blocking and agonist activities of anti-mPD-1 mAbs, PD-L1^+^ IIA1.6 cells and PD-L1^-^ mFcγRIIB^+^ IIA1.6 cells were used for the co-culture with T cells, respectively. These IIA1.6 cells were pre-treated with mitomycin C (0.2 mg/ml; Fujifilm Wako Pure Chemicals, cat#139-18711) for 2h and were used after wash for 3 times. IFN-γ levels in the culture supernatants after 24h were determined using mouse IFN-γ DuoSet ELISA (R&D Systems, cat#DY485).

### Statistical analysis

Data represent means ± SEM. Statistical significance was calculated by Tukey-Kramer test. *P* values of less than 0.05 were considered significant. Data shown in the figures are representative of two or more experiments that essentially demonstrated similar results.

## Results

### Blocking activities of anti-mPD-1 mAbs

**Figure 1:**
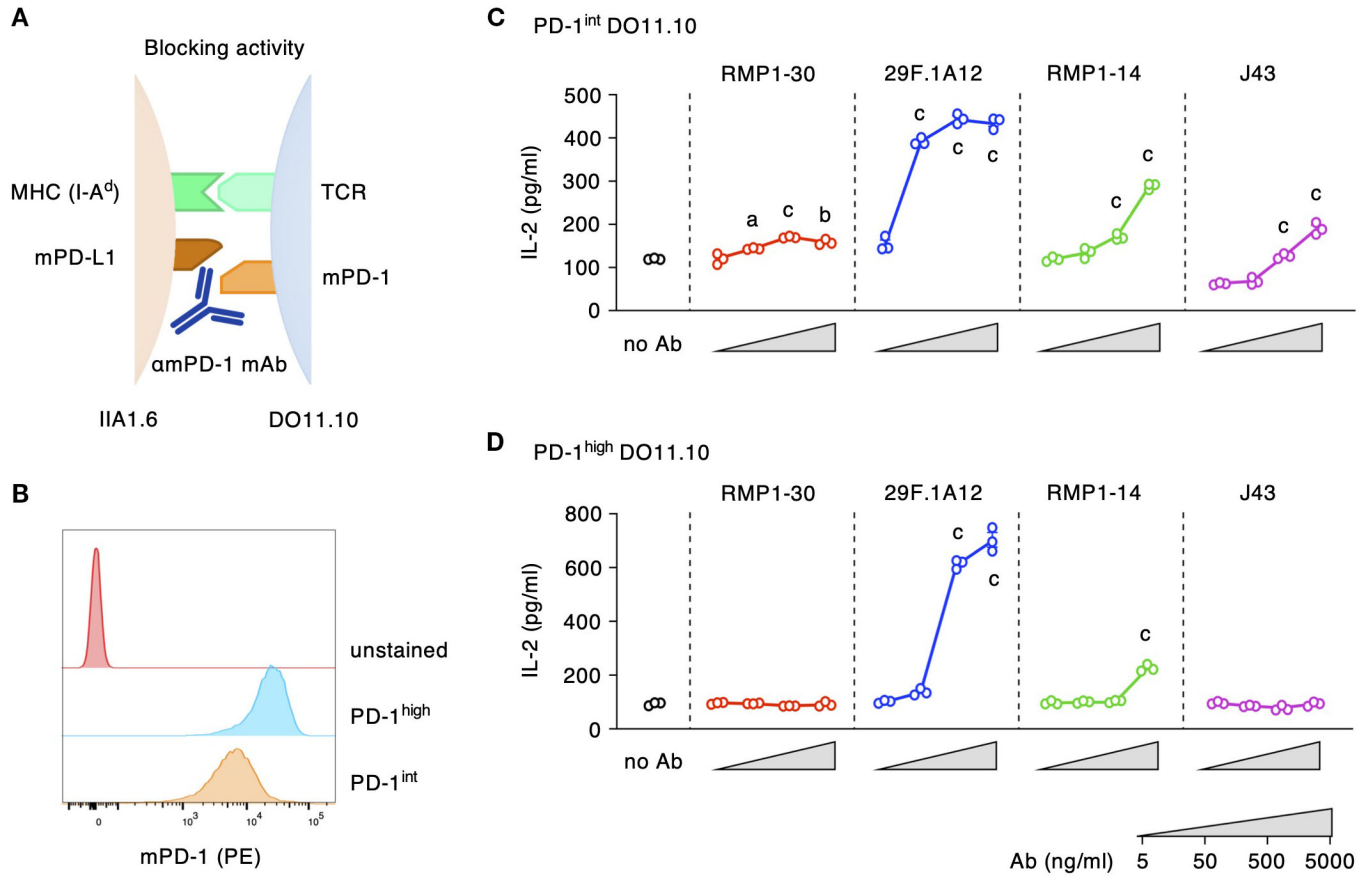
Blocking activities of anti-mPD-1 mAbs. **(A)** Experimental system for the detection of mPD-1 blockade. **(B)** Different levels of mPD-1 expression in PD-1^high^ and PD-1^int^ DO11.10 cells. **(C, D)** Blocking activities of various anti-mPD-1 mAb clones against PD-1^int^ **(C)** and PD-1^high^ DO11.10 cells **(D)**. The reversal of IL-2 inhibition indicates the blockade of PD-1 interaction with PD-L1. Data represents the mean ± SEM (n =3; biological replicates). a, p < 0.05; b, p < 0.01; c, p < 0.001; versus 5 ng/ml; Tukey-Kramer test. Data shown here are representative result of 2 **(C)** and 3 independent experiments **(D)** with the same trend.

We analyzed biological activities of representative commercial anti-mPD-1 mAb clones: 29F.1A12, RMP1-14, J43 and RMP1-30. To evaluate the blocking activity, antibodies were added to the co-culture of PD-1-expressing DO11.10 hybridoma cells and PD-L1-expressing IIA1.6 B lymphoma cells (**Figure 1A**). T cell receptor on DO11.10 cells recognizes its cognate antigen OVA_323–339_ on MHC class II (I-A^d^), but tandem PD-1-PD-L1 interaction inhibits T cell activation and subsequent IL-2 production. Antibodies that can block PD-1-PD-L1 interaction should diminish PD-1-dependent immunosuppression and increase IL-2 production from the T cell line. Since the immunosuppressive activity is associated with PD-1 gene dose (30), we evaluated the biological activities of anti-mPD-1 antibodies using DO11.10 cells with two different expression levels of mPD-1 (**Figure 1B**).

29F.1A12 showed the strongest blocking activity as 50 ng/ml of this antibody significantly increased IL-2 production from PD-1^int^ cells (**Figure 1C**). RMP1–14 and J43 had decent blocking activities, but the reversal of IL-2 reduction was considerably weaker than 29F.1A12. Blocking antibodies constantly performed better to PD-1^int^ cells than to PD-1^high^ cells probably because it would take a large amount of blockers to hinder PD-1 molecules from PD-L1 binding in PD-1^high^ cells. 29F.1A12 still showed a strong blocking activity to PD-1^high^ cells, but the impressive IL-2 increase demanded 500 ng/ml of this antibody (**Figure 1D**). IL-2 increase by RMP1–14 and J43 was only moderate or almost disappeared. RMP1–30 did not indicate a blocking activity. These effects were confirmed to be PD-1-dependent because none of these antibodies increased IL-2 production from PD-1-deficient DO11.10 cells (**Supplementary Figure 2A**).

**Figure 2:**
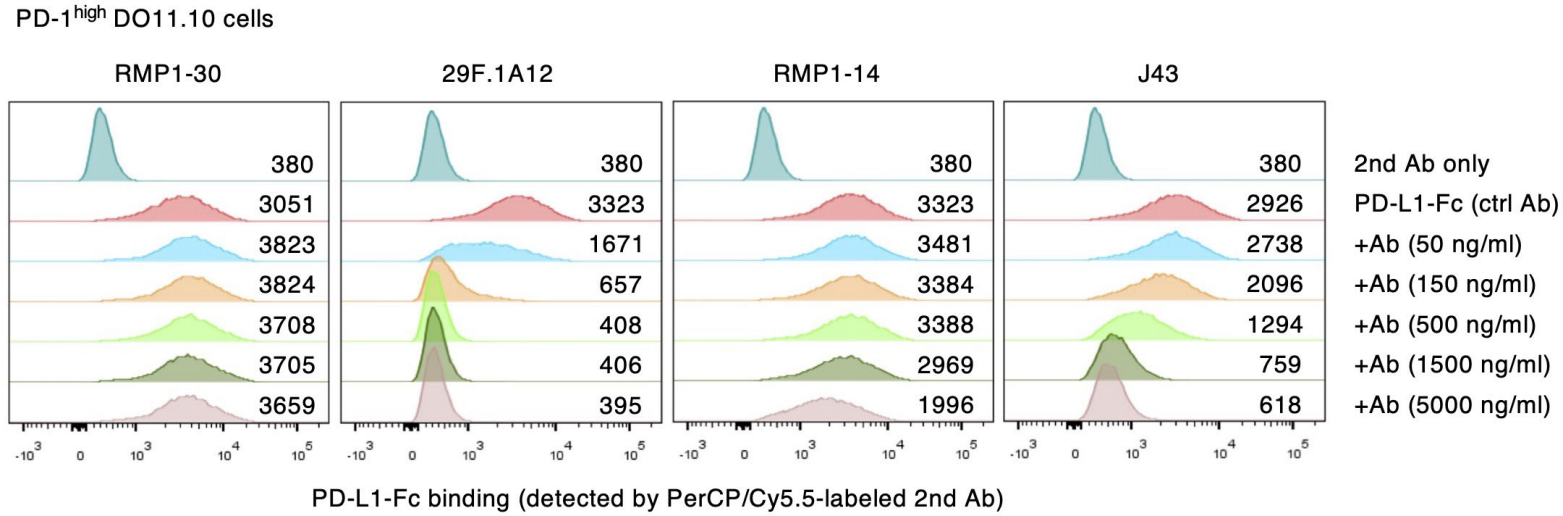
Competitive inhibition of PD-1-PD-L1 interaction by anti-mPD-1 mAbs. PD-1^high^ DO11.10 cells were stained with PD-L1-Fc and PerCP-Cy5.5-labeled secondary antibody. Numbers represent geometric mean fluorescence intensity. Data shown here are representative of 2 independent experiments with the same trend.

Competitive inhibition of PD-L1 binding to PD-1 further confirmed the blocking activities of anti-mouse PD-1 mAb clones. While RMP1–30 did not interfere with PD-L1-Fc binding to PD-1-expressing DO11.10 cells, 50 ng/ml 29F.1A12 reduced PD-L1-Fc binding and completely displaced PD-L1-Fc at higher concentrations (**Figure 2**). The intensities of PD-L1-Fc displacement were weaker with RMP1–14 and J43, which required high concentrations for the incomplete inhibition of PD-L1-Fc binding. The blockade of PD-L1-Fc binding to PD-1^int^ DO11.10 cells indicated the same trend although major reduction of PD-L1-Fc binding was achieved with even lower concentrations of antibodies (**Supplementary Figure 3**). Taken together, 29F.1A12 is the most effective blocking antibody followed by RMP1–14 and J43, but RMP1–30 has no blocking activity. These results are consistent with previous studies in which 29F.1A12 (31, 32), RMP1-14 (33–35) and J43 (36, 37) block PD-L1 binding and promote T cell responses *in vivo*. Meanwhile, RMP1–30 was known not to block PD-L1 binding (31, 33, 34).

### Agonist activities of anti-mPD-1 mAbs

**Figure 3:**
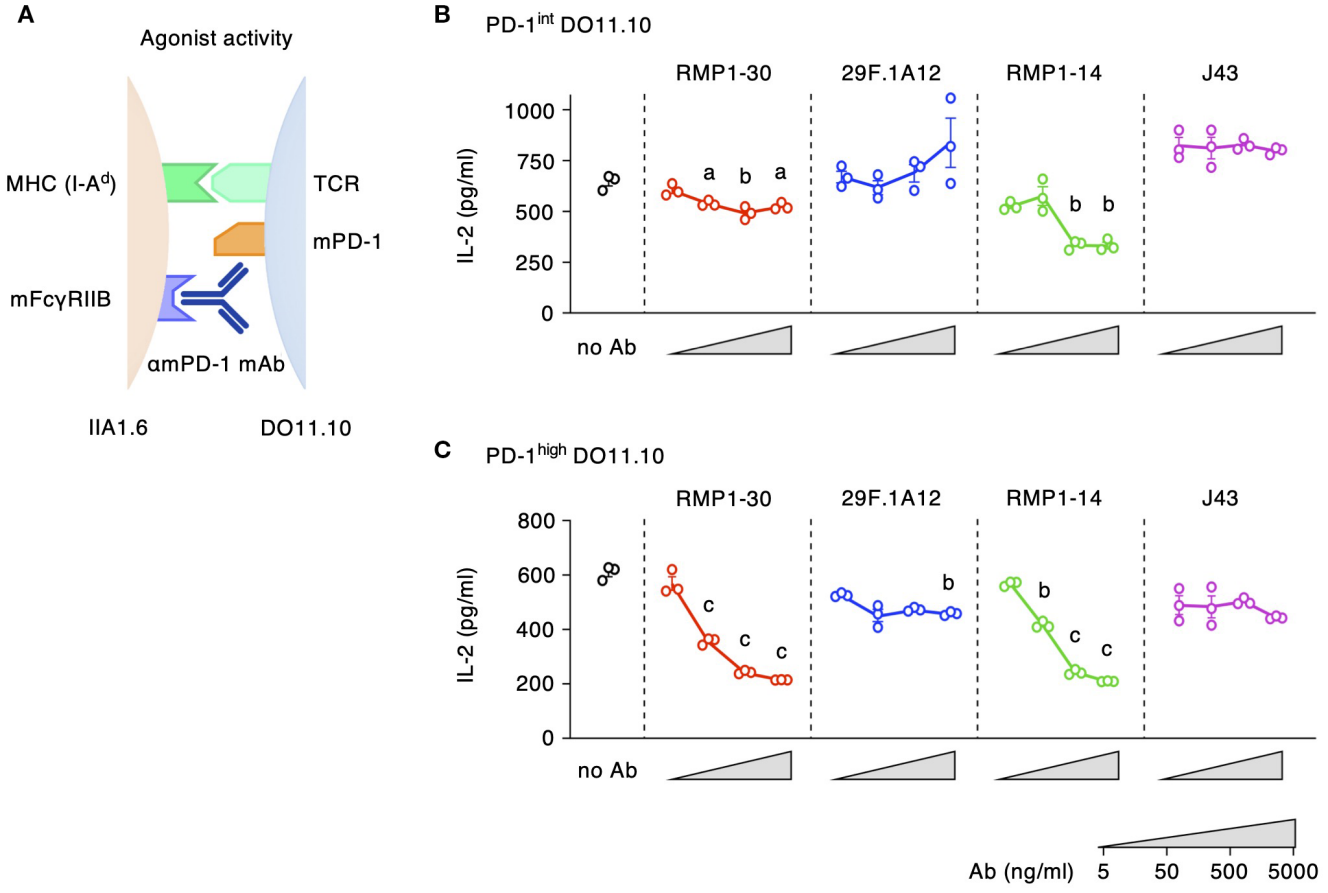
Agonist activities of anti-mPD-1 mAbs. **(A)** Experimental system for the detection of agonistic activity. **(B, C)** Agonistic stimulation by various anti-mPD-1 mAb clones in PD-1^int^ **(B)** and PD-1^high^ DO11.10 cells **(C)**. The extent of IL-2 production indicates the stimulation of immunosuppressive activity by anti-mPD-1 mAbs. Data represents the mean ± SEM (n = 3; biological replicates). a, p < 0.05; b, p < 0.01; c, p < 0.001; versus 5 ng/ml; Tukey-Kramer test. Data shown here are representative of 5 independent experiments with the same trend.

The agonist activity of anti-mPD-1 mAbs was analyzed by the reduction of IL-2 production from DO11.10 cells. IIA1.6 cells for this assay were lacking PD-L1 but were transduced with FcγRIIB to enable PD-1 crosslinking by the antibodies (27) (**Figure 3A**). This assay identified agonist activities in RMP1–30 and RMP1-14 (**Figures 3B, C**). The inhibition of IL-2 production by these antibodies intensified in a dose-dependent manner reaching 60% inhibition in PD-1^high^ cells. 29F.1A12 showed minor IL-2 reduction, but J43 did not significantly decrease IL-2 levels. The use of PD-1^int^ cells diminished the sensitive detection of PD-1 agonist activity. IL-2 inhibition by RMP1–30 and RMP1–14 was 20-40% in PD-1^int^ cells, and no inhibitory effect was detectable for 29F.1A12. Such agonist activities were not observed when applied to PD-1-deficient DO11.10 cells (**Supplementary Figure 2B**). Thus, our screening identified RMP1–30 as mPD-1 agonist antibody. RMP1–14 was found to be unique for having both agonist and blocking activities.

**Figure 4:**
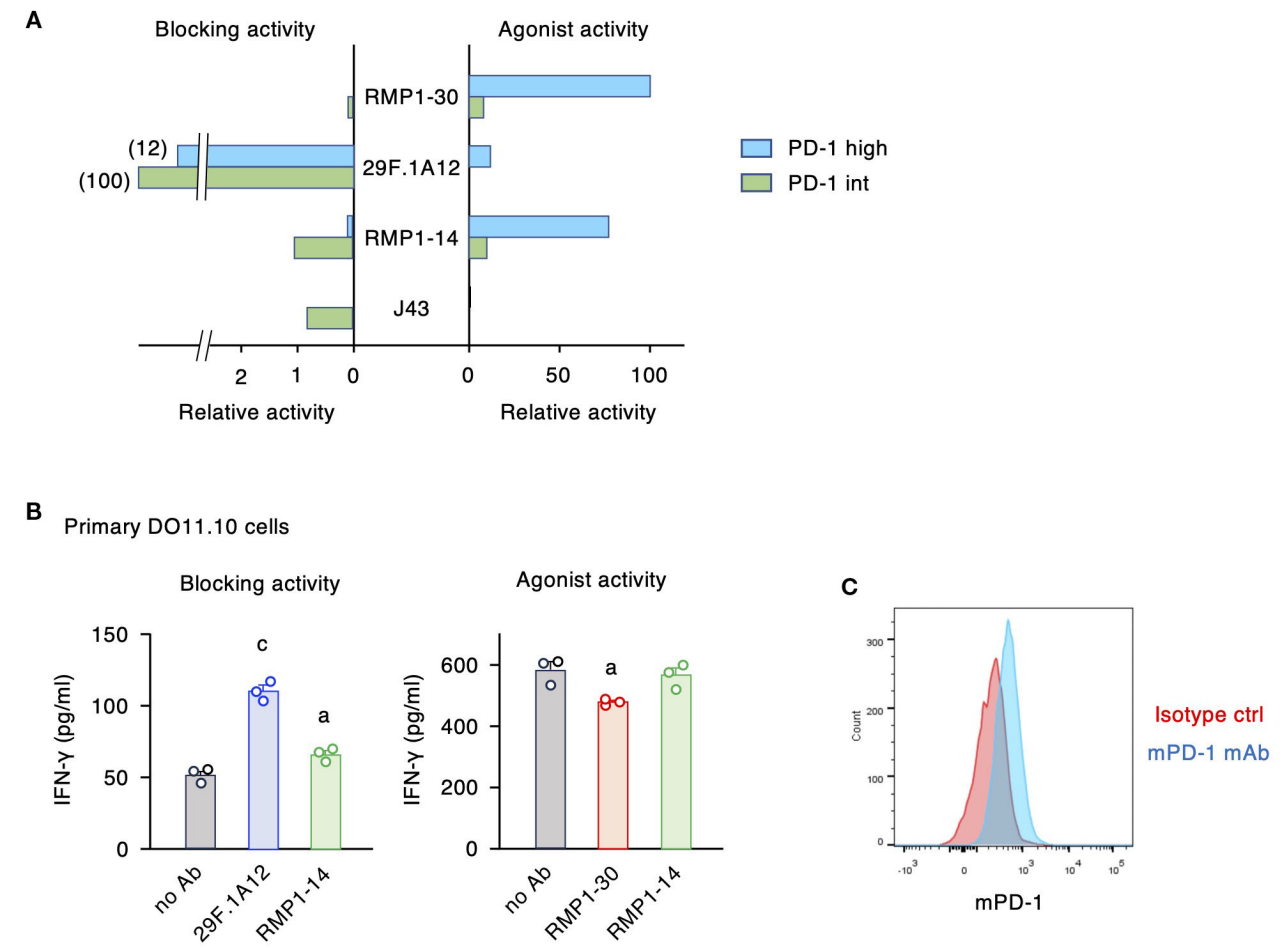
PD-1 expression levels critically affect the intensities of blocking and agonist activities. **(A)** The relative activities were calculated from the 20% effective antibody concentration in the blocking assay and the 10% inhibitory antibody concentration in the agonist assay. The inverse numbers of EC20 and IC10 were converted to the percentages to the highest activities. **(B)** Biological activities of anti-mPD-1 mAbs in primary CD4^+^ T cells. Activated CD4^+^ T cells from DO11.10 T cell receptor-transgenic mice were restimulated with 100 μg/ml ovalbumin in the presence of PD-L1^+^ IIA1.6 cells (blocking activity) or PD-L1^-^ FcγRIIB^+^ IIA1.6 cells (agonist activity). Concentration of antibodies was 5 μg/ml. IFN-γ levels by control rat IgG2a in the blocking assay was 84 ± 7% of no Ab. In the agonist assay, IFN-γ levels in the presence of control rat IgG2a and rat IgG2b were 101 ± 8% and 97 ± 7% of no Ab, respectively. RMP1–14 and 29F.1A12 are rat IgG2a, and RMP1–30 is rat IgG2b. **(C)** PD-1 expression in activated CD4^+^ T cells from DO11.10 T cell receptor-transgenic mice on day 8. Data represents the mean ± SEM (n = 3; biological replicates). a, p < 0.05; c, p < 0.001; versus no Ab; Tukey-Kramer test. Data shown here are representative of 2 independent experiments with the same trend.

The intensity of PD-1-dependent T cell inhibition is positively associated with the levels of PD-1 expression (27, 30). When PD-1-transduced cell lines are utilized to evaluate blocking and agonistic activities, the levels of PD-1 expression considerably affect the assay sensitivities. Specifically, agonist activities weakened as PD-1 expression lowered, while the blocking activities became more notable (**Figure 4**). The agonistic activity of RMP1–30 and RMP1–14 was quite notable in PD-1^high^ cells but was diminished when PD-1 expression was reduced. In contrast, PD-1^int^ cells were the sensitive detectors of PD-1 blocking mAbs compared to PD-1^high^ cells, since a smaller amount of blockers would sufficiently neutralize the immunosuppressive signaling in PD-1^int^ cells. The dual activities of RMP1–14 shifted the balance favoring blocking activity as the PD-1 expression level was reduced (**Figure 4A**).

To examine the predominant role of dual-functional RMP1–14 at normal PD-1 levels, we used primary-cultured CD4^+^ T cells in place of PD-1-transduced cell lines. Anti-mPD-1 mAbs were added at the time of restimulation of activated T cells where the blocking and agonist activities were evaluated using PD-L1^+^ IIA1.6 cells and PD-L1^-^ mFcγRIIB^+^ IIA1.6 cells as antigen-presenting cells, respectively. 29F.1A12 clearly reversed the suppressed production of IFN-γ by PD-L1-expressing antigen-presenting cells, and RMP1–14 could also increase cytokine production to a lesser degree (**Figure 4B**). For the agonist activity, RMP1–30 showed a moderate but significant reduction of IFN-γ production, while RMP1–14 failed to do so. Such responses of primary T cells resemble that of PD-1^int^ cell line in consistence with the moderate PD-1 expression in these T cells (**Figure 4C**). RMP1–30 will function as an agonist *in vivo* although the intensity of immunosuppression might be variable dependent on PD-1 expression levels. RMP1–14 is more likely to act as a blocker when injected to mice, and this prediction is consistent with the previous reports of improved tumor regression by RMP1-14 (35, 38, 39).

### Binding sites of anti-mPD-1 agonist mAbs

**Figure 5:**
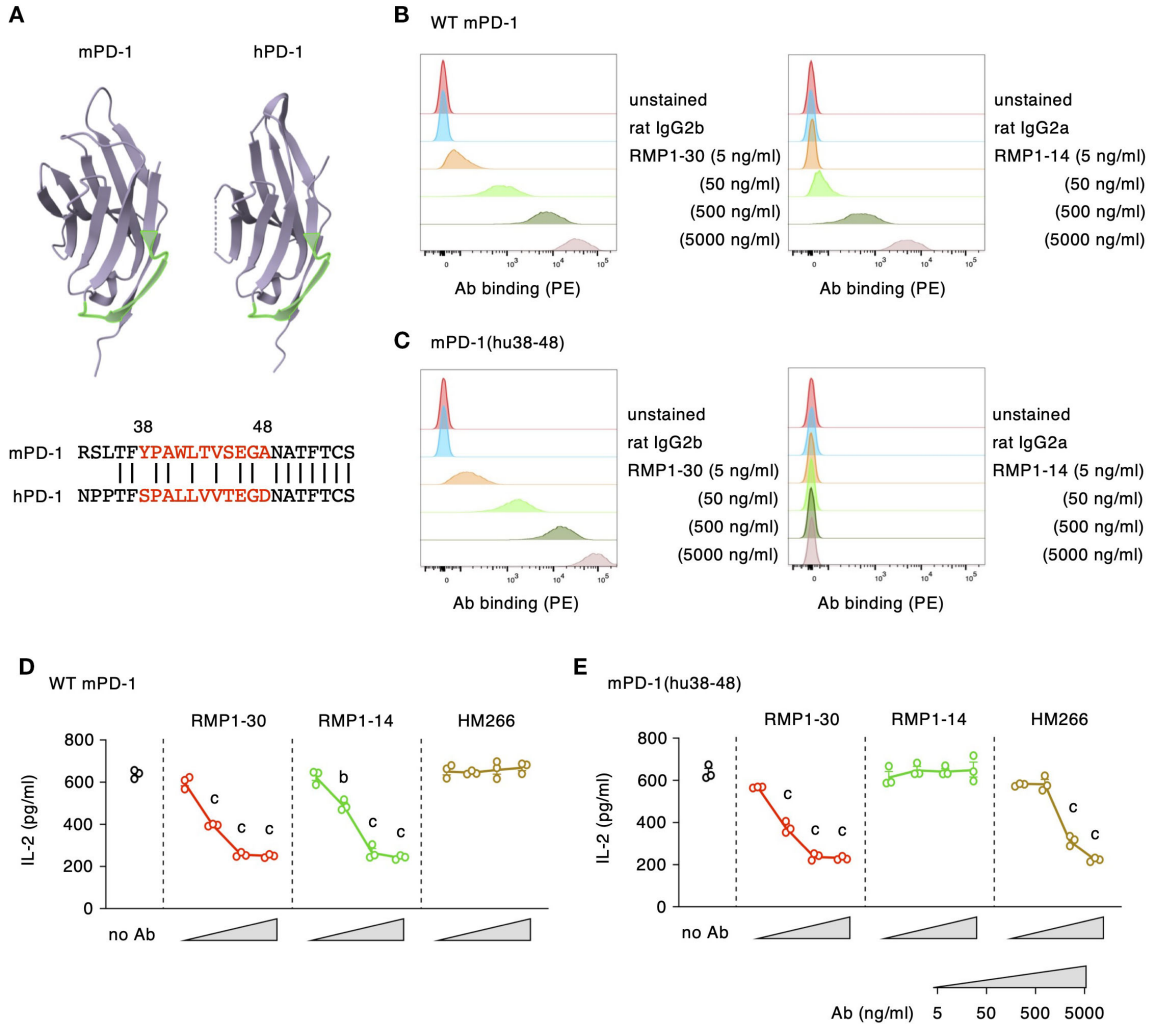
RMP1-14, but not RMP1-30, induces the agonist activity through the recognition of mPD-1_38–48_ segment. **(A)** The putative membrane-proximal localization of mPD-1_38–48_ and hPD-1_38-48_ (shown in light green). The 3D models of the extracellular domains were drawn using the RCSB Protein Data Bank web site (https://www.rcsb.org; mPD-1, 1NPU; hPD-1, 3RRQ). **(B, C)** Antibody bindings to DO11.10 cells expressing wild-type mPD-1 **(B)** or mPD-1(hu38-48) **(C)**. **(D, E)** PD-1 agonist activities in DO11.10 cells expressing wild-type mPD-1 **(D)** or mPD-1(hu38-48) **(E)** at high levels. HM266 is anti-hPD-1 agonist mAb recognizing the hPD-1_38–48_ segment. Data represents the mean ± SEM (n = 3; biological replicates). b, p < 0.01; c, p < 0.001; versus 5 ng/ml; Tukey-Kramer test. Data shown here are representative of 2 independent experiments with the same trend.

Our previous study on anti-hPD-1 antibodies characterized agonists as a distinct group of antibodies from blockers in terms of their binding domain (27). PD-1 blocking antibodies found their binding sites close to the PD-L1-binding domain, which is distal from the plasma membrane. In contrast, PD-1 agonist antibodies specifically recognized the membrane-proximal region, especially the recognition of hPD-1_38–48_ segment was associated with strong agonistic activity. To examine the similar membrane-proximal recognition by mPD-1 agonist antibodies, we prepared mutant mPD-1 with hPD-1_38–48_ replacing the corresponding mPD-1_38–48_ sequence (**Figure 5A**). The mPD-1_38–48_ segment is located at the membrane-proximal region similar to hPD-1_38-48_. RMP1–14 at concentrations that displayed apparent binding to wild-type mPD-1 lost its binding to DO11.10 cells expressing the mutant mPD-1(hu_38-48_), suggesting that RMP1–14 recognizes the membrane-proximal mPD-1_38–48_ segment (**Figures 5B, C**). In contrast, the replacement of mPD-1_38–48_ with hPD-1_38–48_ did not affect RMP1–30 binding at all. Since no binding reduction was observed even at the lowest concentration of antibody, RMP1–30 is likely to induce agonist activity through the binding to other domain than mPD-1_38-48_.

We further examined the capability of mPD-1 agonist antibodies to downregulate IL-2 production from mPD-1(hu_38-48_)-expressing DO11.10 cells. Consistent with the binding characteristics, RMP1–30 could suppress IL-2 production from wild-type mPD-1- and mPD-1(hu_38-48_)-expressing cells to the same degree, whereas RMP1–14 did not reduce IL-2 from mPD-1(hu_38-48_)-expressing cells (**Figures 5D, E**). HM266 is one of the anti-hPD-1 agonist mAbs recognizing hPD-1_38–48_ segment. The immunosuppressive effect of HM266 to mPD-1(hu_38-48_)-expressing DO11.10 cells, but not wild-type mPD-1, confirmed that the binding of HM266 particularly at the hPD-1_38–48_ domain was sufficient to induce agonistic activity.

### Fc receptor requirement for anti-mPD-1 agonist mAbs

**Figure 6:**
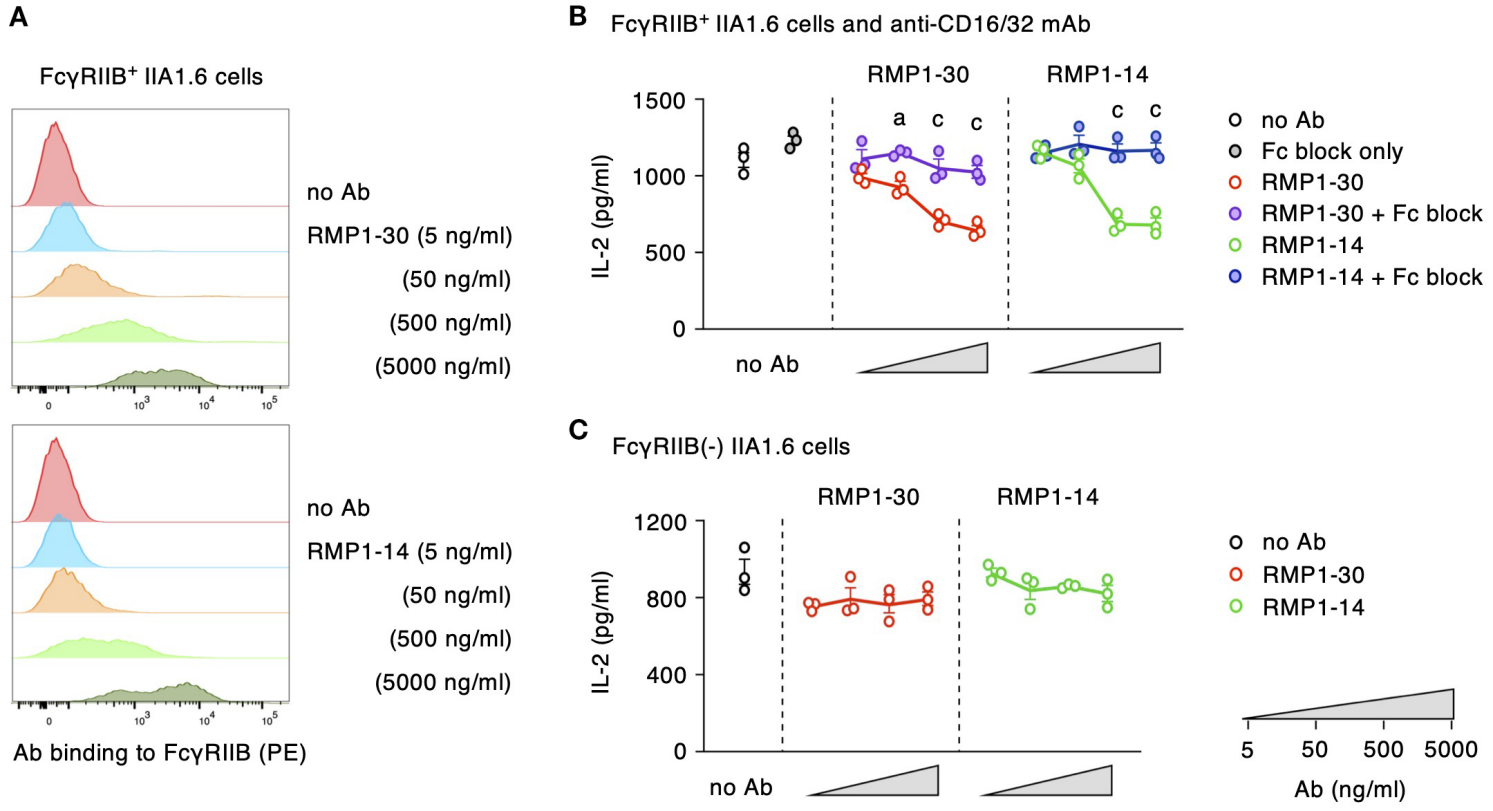
RMP1–30 and RMP1–14 demand Fc receptors for the induction of agonist activity. **(A)** Antibody binding to FcγRIIB-transduced IIA1.6 cells. **(B)** Agonist activity of anti-PD-1 mAbs and its reversal by the blockade of Fc receptor. Fc block (anti-CD16/32 mAb) was added at 10 μg/ml. **(C)** RMP1–30 and RMP1–14 did not indicate their agonist activity in the co-culture with IIA1.6 cells lacking Fc receptors. PD-1^high^ DO11.10 cells were used in these experiments. Data represents the mean ± SEM (n = 3; biological replicates). a, p < 0.05; c, p < 0.001; versus anti-mPD-1 mAb alone; Tukey-Kramer test. Data shown here are representative of 3 independent experiments with the same trend.

In addition to the binding site, our previous study on anti-hPD-1 mAbs showed another requirement for PD-1 agonists, crosslinking of PD-1 molecules through the engagement to Fc receptors (27). Next, we examined whether RMP1–30 and RMP1–14 share the same requirement as hPD-1 agonist mAbs or not. Both RMP1–30 and RMP1–14 were confirmed to interact with mouse FcγRIIB where RMP1-30, rat IgG2b, showed slightly stronger binding compared to RMP1-14, rat IgG2a (**Figure 6A**). In the induction of PD-1 agonist activity, the addition of anti-CD16/32 mAb to block the interaction with FcγRIIB reversed IL-2 reduction by RMP1–30 and RMP1-14 (**Figure 6B**). The requirement of Fc receptor engagement for the agonist activity was further confirmed in the co-culture with IIA1.6 cells lacking Fc receptor expression. RMP1–30 and RMP1–14 did not inhibit IL-2 production in the absence of Fc receptors (**Figure 6C**). J43 (hamster IgG) could bind to mouse FcγRIIB but did not exert the agonist activity (**Supplementary Figure 4**). Anti-mPD-1 agonist mAbs trigger the immunosuppressive activity upon binding to the membrane-proximal region of PD-1 on T cells and Fc receptor engagement on antigen-presenting cells.

## Discussion

In the current study, we characterized biological activities of 4 commercial anti-mPD-1 mAbs and found agonistic potential in RMP1–30 and RMP1-14 (summarized in **Table 1**). Antibodies to cell-surface receptors often trigger the agonist activity by the Fc receptor- mediated crosslinking of antibody-bound target receptors, e.g. CD3 and TNF receptor family members including CD40 (40–42). Agonist antibodies to PD-1 also demanded Fc receptor-mediated crosslink for the induction of immunosuppressive activity. RMP1–30 and RMP1–14 are rat IgG, which is not ideal for the binding to mouse Fc receptors (43). Since RMP1–14 is dual-functional, RMP1–30 will be a choice if pharmacological PD-1 stimulation is needed; however, the intensity of PD-1 stimulation seems to be moderate in primary mouse T cells (**Figure 4B**). In our previous study, anti-hPD-1 agonist mAb in the normal human IgG form showed only limited immunosuppressive activity against primary human T cells, but Fc engineering to increase the affinity to human FcγRIIB strongly enhanced the agonist activity (27). The optimization of Fc region of RMP1–30 to enhance Fc receptor engagement may intensify the agonist activity and expand the utility as a research tool.

**Table 1:** Biological activities of anti-mouse PD-1 antibodies.

Clone	Blocking		Agonist		
	Activity	PD-L1 competition	Activity	Fc receptor requirement	mPD-1_38-48_ binding
RMP1-30	–	–	+	yes	no
RMP1-14	+	(+)	+*	yes	yes
J43	+	+	–		
29F.1A12	++	++	–		

*Agonistic potential of RMP1–14 was evident in PD-1-overexpressing T cell lines but not primary-cultured CD4^+^ T cells.

Many antibodies that bind to the membrane-proximal region of PD-1 molecule were agonists in our previous screening of hPD-1 mAb panel (27). In the current study, RMP1–14 induced agonist activity through the binding to mPD-1_38–48_ at the membrane-proximal region. RMP1–30 belongs to a different group of agonists recognizing a separate domain from mPD-1_38-48_. Previous studies have shown that RMP1–30 does not recognize PD-L1 binding site (33, 34) or compete with RMP1-14, 29F.1A12 or J43 for PD-1 binding (25, 31). RMP1–30 represents PD-1 agonist mAbs with a unique binding site, but its apparent lack of blocking ability implies the recognition of membrane-proximal part of the mPD-1 molecule.

Among agonist mAbs to hPD-1 and mPD-1, what is uncommon with RMP1–14 is the co-existing blocking capability. The reason for this unusual duality of RMP1–14 functions remains unclear. The binding property of RMP1–14 might be unconventional for a blocking antibody. PD-L1 displacement by RMP1–14 was relatively weak compared with J43 although these antibodies showed similar levels of blocking activity (**Figures 1**, **2**). RMP1–14 does not interfere with the PD-1 binding of other blocking antibodies (25, 31) and binds to the membrane-proximal mPD-1_38–48_ instead (**Figure 5**). Such a membrane-proximal binding would have contributed to the acquisition of agonist activity, but it will be interesting to know how RMP1–14 can be so functional as a blocking antibody. Weak agonist activity was also detectable with 29F.1A12, an outstanding blocking antibody among 4 tested clones (**Figure 4**). Interestingly, recent papers reported agonistic potentials in the therapeutic PD-1 blockers, nivolumab and pembrolizumab (28, 29). Dual functions may be present in RMP1-14, 29F.1A12, nivolumab and pembrolizumab; however, all of these antibodies do enhance anti-tumor immune response, indicating that they are substantially blockers *in vivo*.

To explain why agonist activities can be observed in these PD-1 blocking mAbs, we speculate the influence of PD-1 expression levels in the assay system. In this study, we used mPD-1-transduced cell line and found that excessive PD-1 expression can result in overly sensitive detection of agonist activity (**Figure 4A**). RMP1–14 appeared to be an agonist antibody when applied to PD-1^high^ cells. However, the same antibody behaved as a blocking antibody in PD-1^int^ cells. Furthermore, RMP1–14 showed decent blocking activity, but no detectable agonist activity, in primary-cultured T cells at least in this experimental setting (**Figure 4B**). Considering PD-1 levels in T cells during immune response, anti-PD-1 antibodies with the dual potential will primarily act as blockers *in vivo*. Retrospectively, we did not observe any dual-function clone in our previous screening of anti-hPD-1 mAbs probably due to the use of intermediate PD-1 levels (27). Thus, PD-1 expression levels would be an important factor in evaluating physiologically-relevant outcomes.

**Figure 7:**
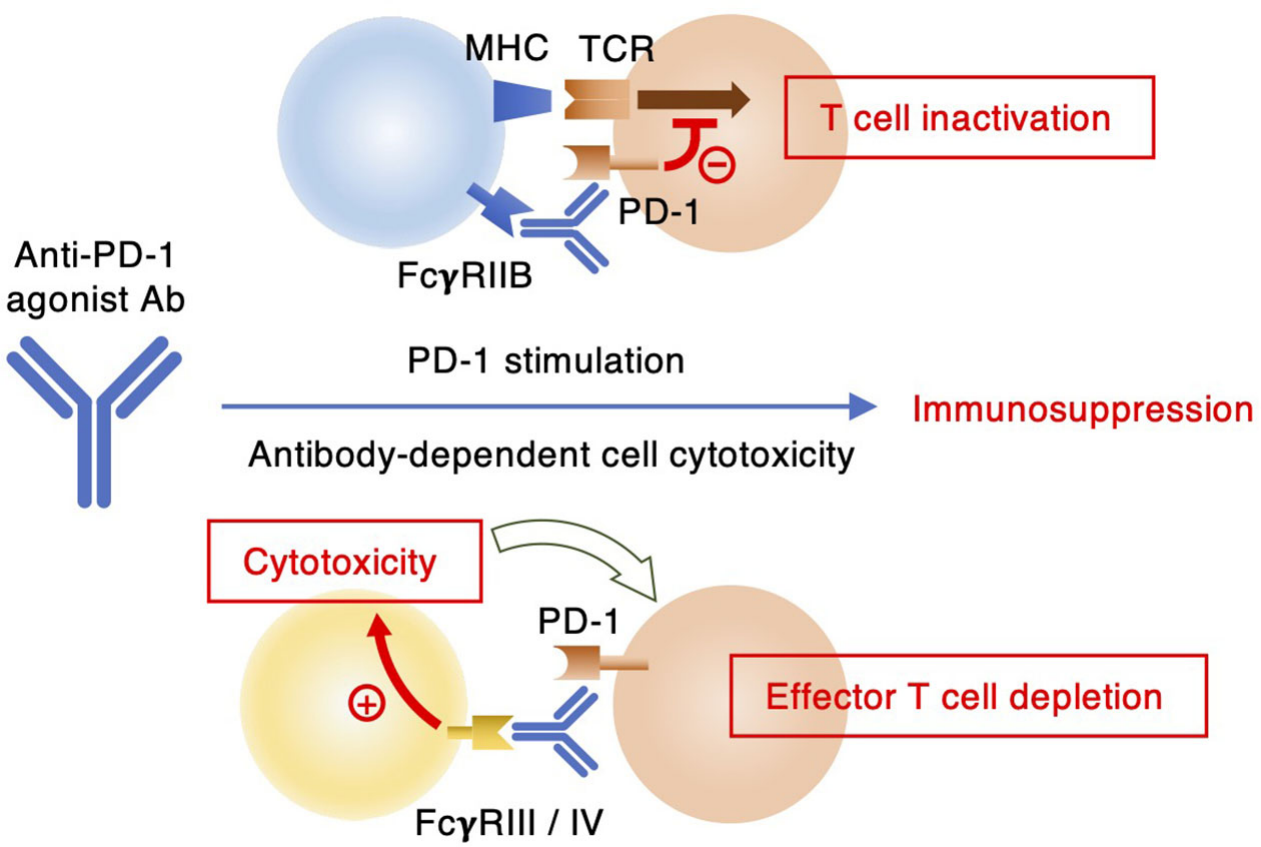
Two different mechanisms of immunosuppression by anti-PD-1 antibodies. Agonist antibodies can interrupt T cell receptor signaling through FcγRIIB-mediated PD-1 stimulation. Some anti-PD-1 antibodies can exert depletion of PD-1-expressing immune effector cells by ADCC upon interaction with FcγRIII or FcγRIV.

Although their primary role is PD-1 blockade, it is still possible that the agonistic potential of PD-1 blocking antibodies may adversely affect the anti-tumor efficacy. RMP1–14 was previously found to be more effective in suppressing tumor growth in FcγRIIB-deficient mice than in wild-type controls (35). While the absence of immunosuppressive FcγRIIB signaling might have played a role in enhancing anti-tumor immunity (44, 45), they speculated right about the presence of agonistic potential in RMP1-14. Apart from the agonist activity, Fc receptor binding of PD-1 blocking antibody can be detrimental to anti-tumor immunity through the removal of effector T cells by antibody-dependent cell cytotoxicity and/or complement-dependent cytotoxicity (**Figure 7**). In cancer immunotherapy, Fc receptor-dependent deletion of PD-1-expressing effector T cells compromises anti-tumor response as evidenced from the improved anti-tumor efficacy by the elimination of Fc receptor binding (25, 35, 46). Clinical PD-1 blocking antibodies such as nivolumab and pembrolizumab are provided in human IgG4 form, which does not induce significant antibody-dependent cell cytotoxicity due to its low affinity to human FcγRIIIA. The reduction of Fc receptor binding may enhance the efficacy of PD-1 blocking antibodies in cancer immunotherapy by preventing the elimination of anti-tumor effector cells and the induction of immunosuppressive PD-1 signaling.

In conclusion, we identified RMP1–30 as anti-mPD-1 agonist antibody. The requirement of Fc receptor-mediated crosslink was common to anti-hPD-1 agonist mAbs. Anti-mPD-1 agonist antibody will be useful for the application in various murine disease models. Its anti-inflammatory efficacy *in vivo* may vary dependent on Fc receptor expression in the local tissue environment, PD-1 expression levels on therapeutic targets and type of inflammation. It should be noted that RMP1–30 is rat IgG2b, which can extensively deplete antibody-bound cells in mice. Although the removal of proinflammatory effector cells will not compromise the use of anti-PD-1 mAb for anti-inflammatory purpose, *in vivo* application of RMP1–30 may involve conceptually distinct mechanisms: PD-1 stimulation and effector cell depletion (**Figure 7**). At this time, it will be difficult to specify the mechanism of immunosuppressive outcome due to technical limitation. Fc engineering of RMP1–30 will help distinguish these two possible mechanisms of immunosuppression. RMP1–14 offers both agonist and blocking activities. While RMP1–14 has been useful as a PD-1 blocker in various tumor models, this dual-function antibody may have a chance to evoke the agonist activity dependent on PD-1 expression levels and Fc receptor availability. In this sense, 29F.1A12 will be more straightforward than RMP1–14 for PD-1 blocking purpose. These interpretations are yet to be validated in murine models of inflammatory diseases and cancer.

## Data Availability

The raw data supporting the conclusions of this article will be made available by the authors, without undue reservation.
