# Supplementary figures and images for: Identification of anti-mouse PD-1 agonist antibodies that inhibit T cell activation
Source: Front Immunol. 2025 Sep 23;16:1631929. doi: 10.3389/fimmu.2025.1631929 (PMC12500435; doi:10.3389/fimmu.2025.1631929)

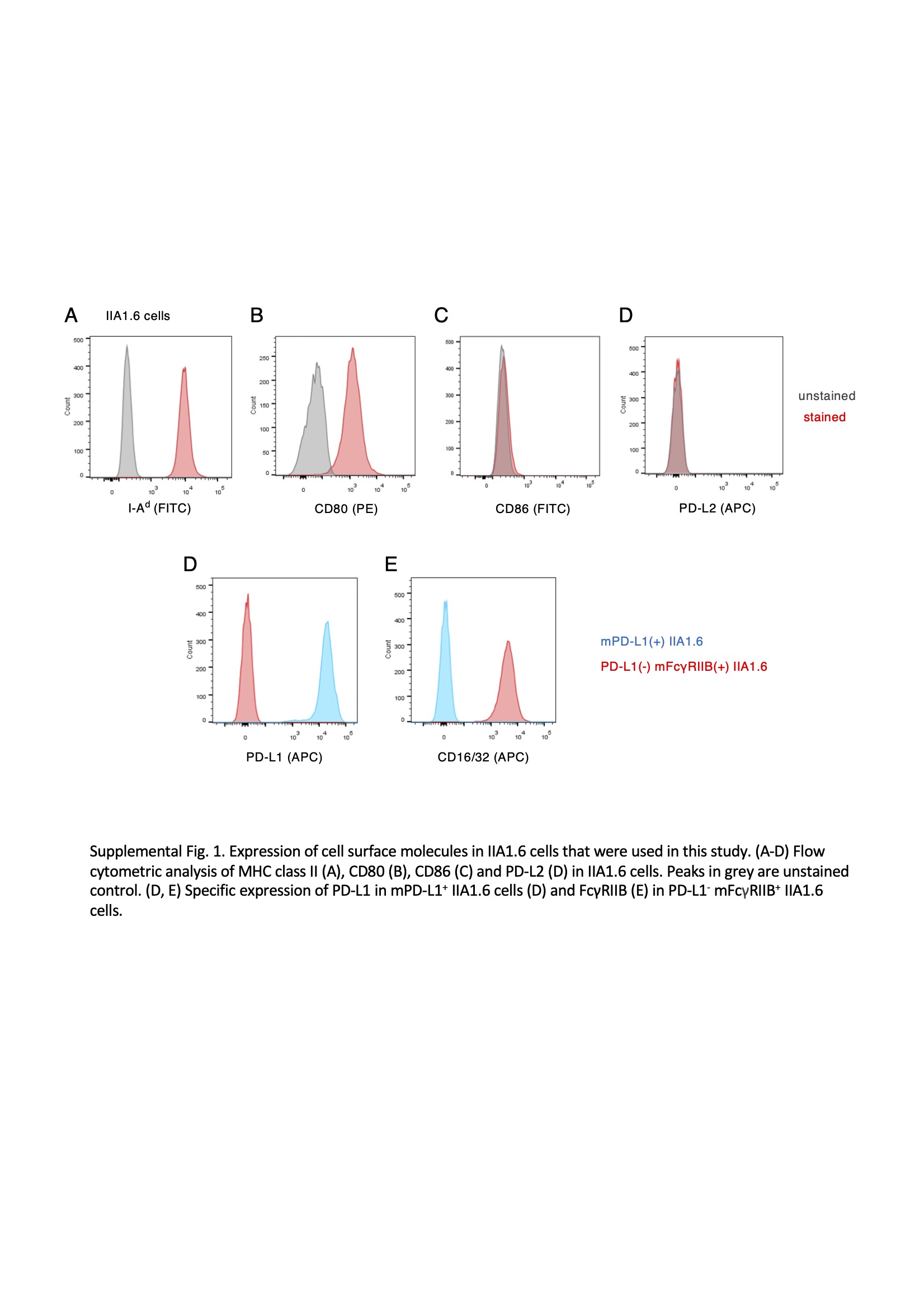

Supplement: Supplementary file 1 [file Image1.jpeg]

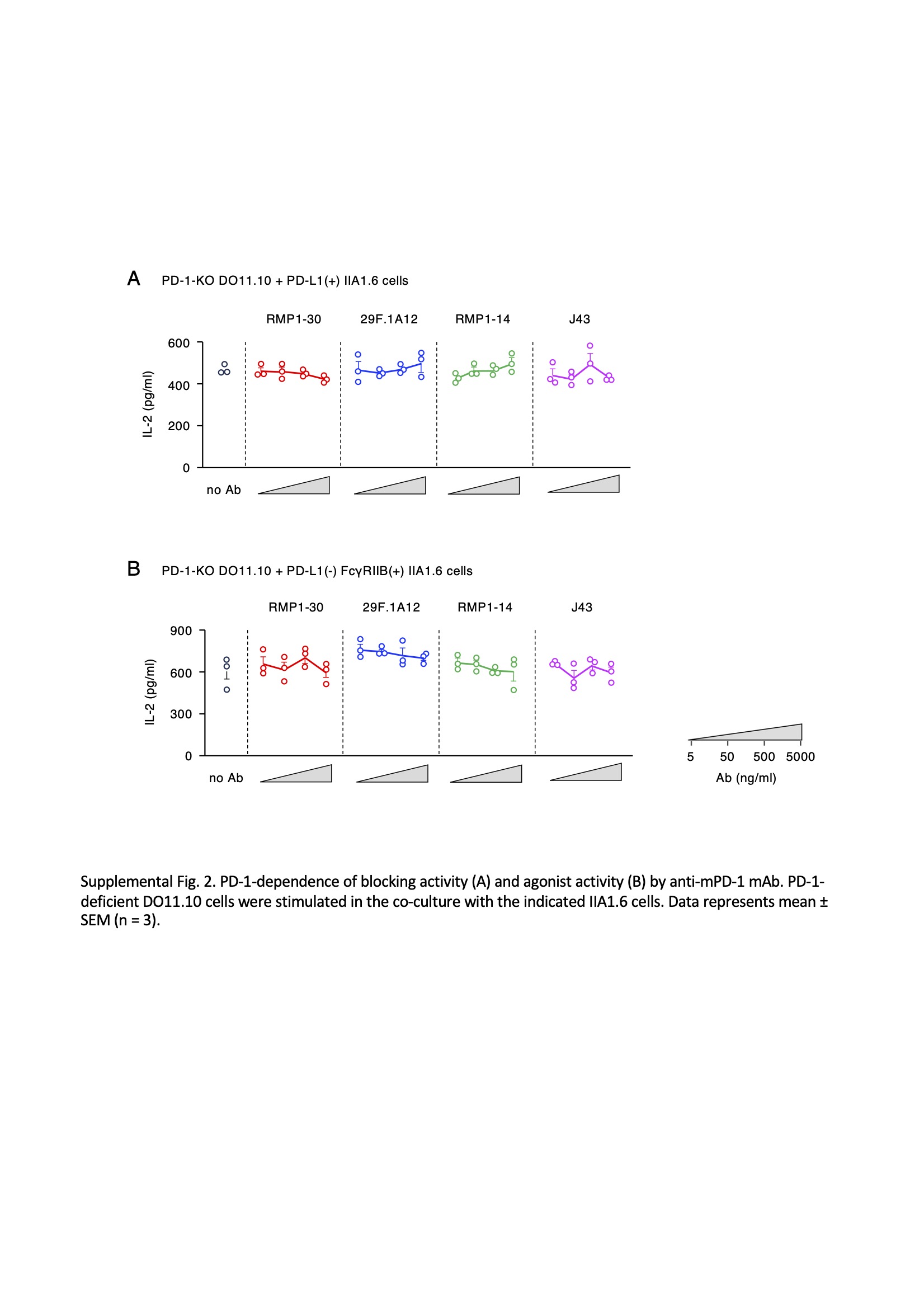

Supplement: Supplementary file 2 [file Image2.jpeg]

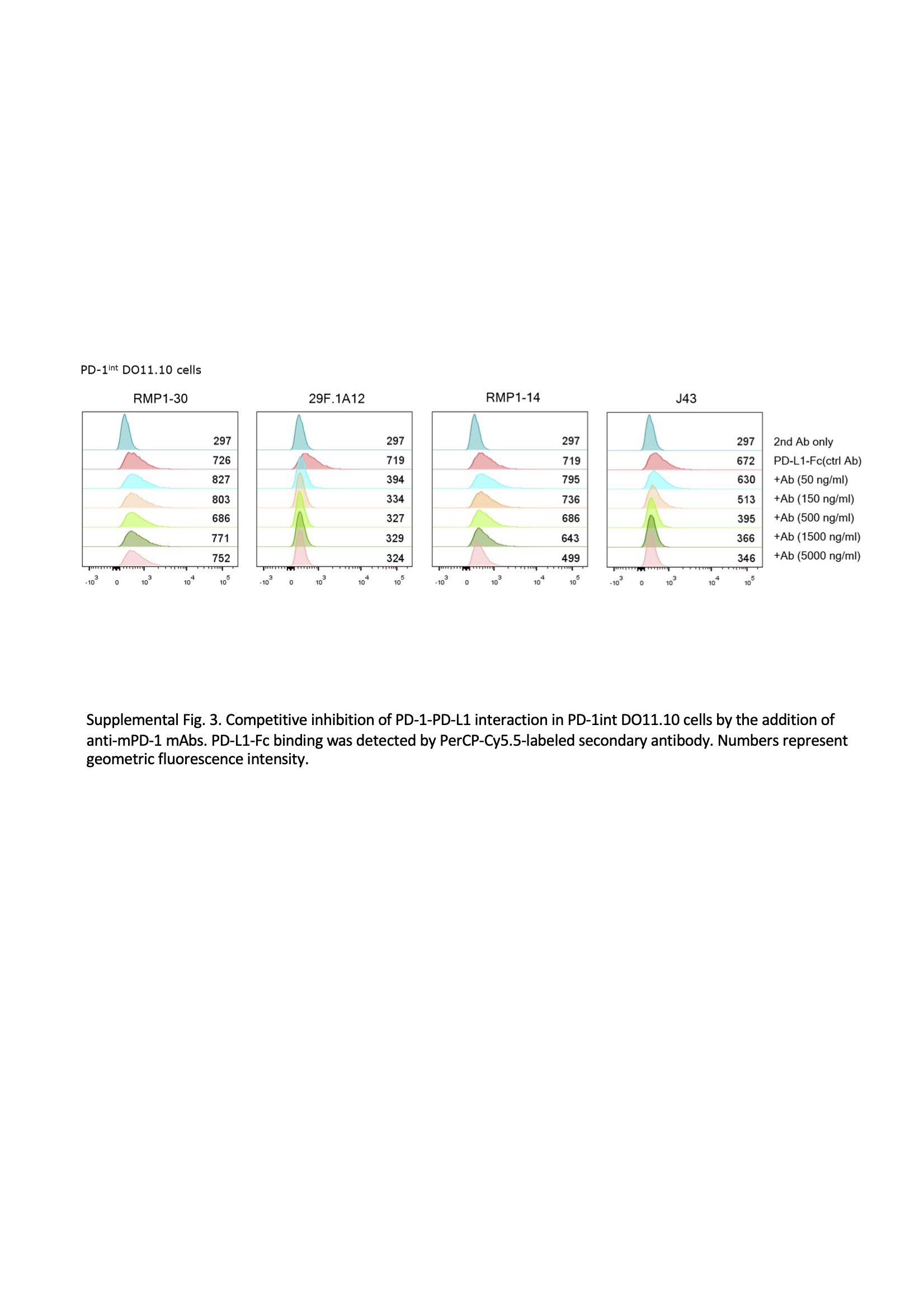

Supplement: Supplementary file 3 [file Image3.jpeg]

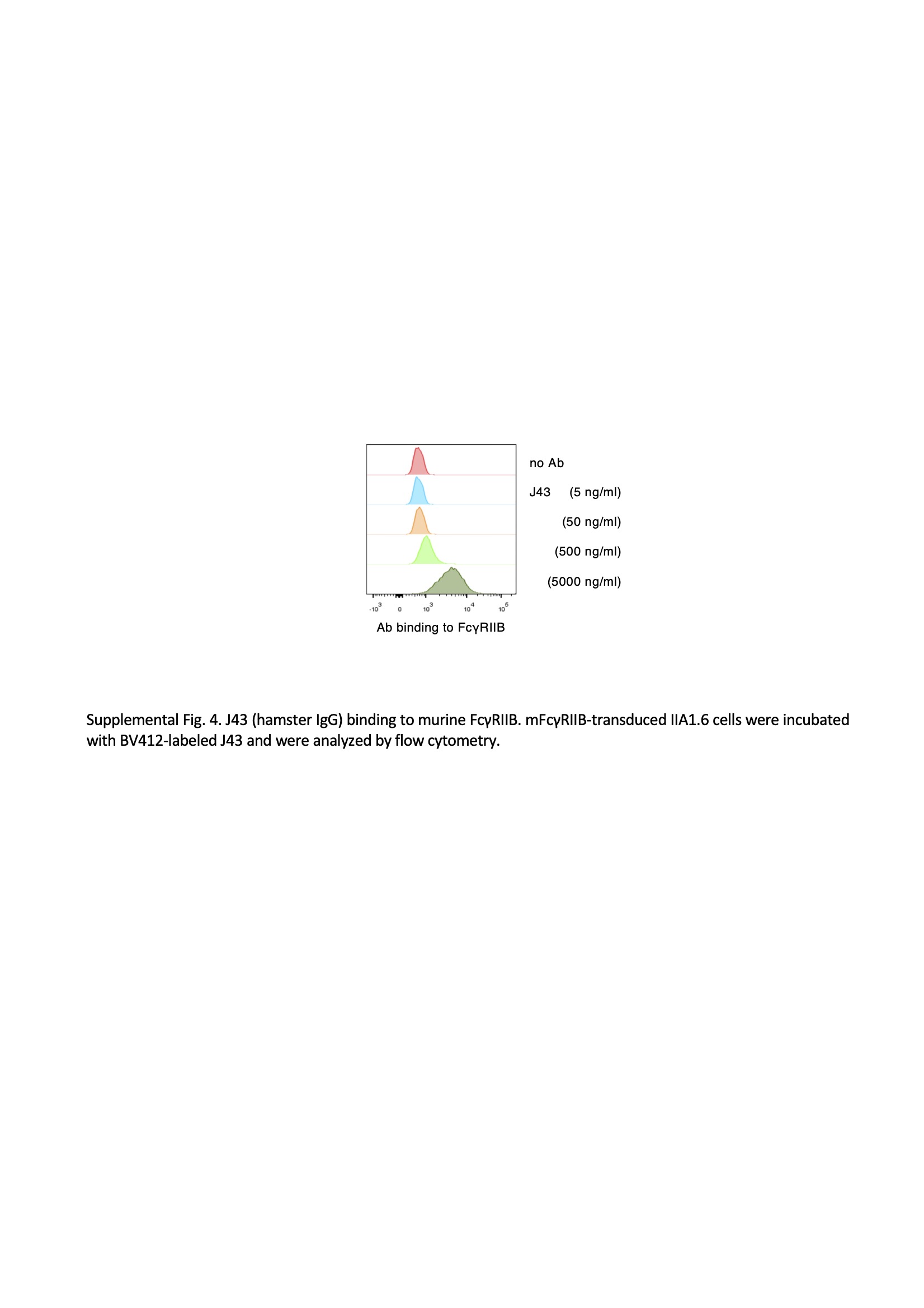

Supplement: Supplementary file 4 [file Image4.jpeg]
